# The Elasticity of Polymer Melts and Solutions in Shear and Extension Flows

**DOI:** 10.3390/polym15041051

**Published:** 2023-02-20

**Authors:** Andrey V. Subbotin, Alexander Ya. Malkin, Valery G. Kulichikhin

**Affiliations:** 1 A.V. Topchiev Institute of Petrochemical Synthesis, Russian Academy of Sciences, Leninskii prosp. 29, Moscow 119991, Russia; 2 A.N. Frumkin Institute of Physical Chemistry and Electrochemistry, Russian Academy of Sciences, Leninskii prosp. 31, Moscow 119071, Russia

**Keywords:** polymers, viscoelasticity, shear, extension, jet

## Abstract

This review is devoted to understanding the role of elasticity in the main flow modes of polymeric viscoelastic liquids—shearing and extension. The flow through short capillaries is the central topic for discussing the input of elasticity to the effects, which are especially interesting for shear. An analysis of the experimental data made it possible to show that the energy losses in such flows are determined by the Deborah and Weissenberg numbers. These criteria are responsible for abnormally high entrance effects, as well as for mechanical losses in short capillaries. In addition, the Weissenberg number determines the threshold of the flow instability due to the liquid-to-solid transition. In extension, this criterion shows whether deformation takes place as flow or as elastic strain. However, the stability of a free jet in extension depends not only on the viscoelastic properties of a polymeric substance but also on the driving forces: gravity, surface tension, etc. An analysis of the influence of different force combinations on the shape of the stretched jet is presented. The concept of the role of elasticity in the deformation of polymeric liquids is crucial for any kind of polymer processing.

## 1. Introduction

Elasticity is obviously an inherent property of polymers. This property determines the huge usage of polymers in rubber industry. In this area, two approaches are naturally combined: the mechanics of large reversible deformations and the physics of interactions and deformations at the molecular level. 

However, what is the level of our understanding of the role of elasticity in the flow of polymer solutions and melts? It is intuitively clear that in shear and tension there is a superposition of reversible and irreversible deformations, which is formulated in many constitutive equations proposed for polymer melts and solutions. Nevertheless, the role of elasticity is important not only for the flow, but for the emergence of new unexpected effects associated with the elastic instability of elastic liquids. Therefore, in this review, we wanted to collect and describe those phenomena that are directly caused by the elasticity of polymeric liquids. At the same time, as in the case of rubbers, we wanted to collect both macroscopic experimental facts and phenomena related to the orientation of individual macromolecules under one roof, which is especially important for the expansion of polymeric liquids.

There are several basic concepts in rheology that form its foundation. These are non-Newtonian flow, viscoelasticity, thixotropy, and viscoplasticity. All of these concepts have been the subject of extensive research, summarized in a large number of monographs and reviews. The elasticity of rheologically complex liquids that is inherent in solutions and melts of polymers to the greatest extent was usually considered a special case of viscoelasticity. However, consideration of the behavior of polymeric liquids demonstrates various effects associated specifically with the elasticity of these media. Next, we will try to review the current state of research in this area, not limited to the effects associated with the elasticity of polymer solutions and melts: the physics of macromolecular deformations responsible for the observed phenomena would also be considered. The analysis of the elasticity of polymeric liquids should be based on the existing fundamental concepts in polymer physics in order to have a common approach to understanding any new experimental fact.

These are:

- The ratio of the scales of internal time and observation time called the Deborah number, De, (Reiner, 1928 [1]), and the ratio of the scales of relaxation rate to deformation rate called the Weissenberg number, Wi [2];
- The consequence of this fundamental approach—the concept of the time (frequency)—temperature superposition approves that the same type of relaxation state (or rheological behavior) can be reached by varying either the rate of deformation (frequency) or temperature (Ferry [3]), Tobolsky [4]);
- The concept of the transition from linear to non-linear mechanical behavior in increasing the deformation rate [5,6].

**Figure 1:**
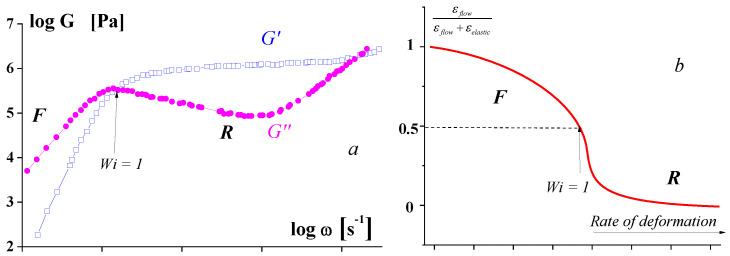
Typical behavior of polymer melts. *a*: relaxation states of polymers: frequency dependencies of the storage *G*′ and loss moduli *G*″, *F*—flow (terminal) and *R*—rubbery states. *b*: ratio between flow and elastic deformations [7].

The following pictures (Figure 1) illustrate these concepts for the domains of linear and non-linear viscoelasticity of polymers.

**Figure 2:**
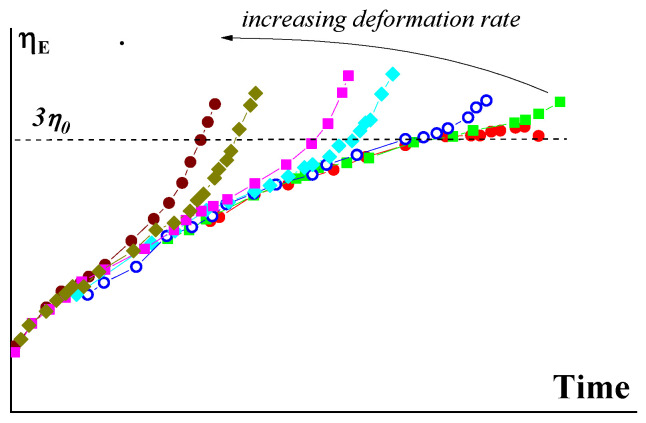
The linear-to-non-linear behavior transition in increasing the deformation rate under extension.

As for the transition to non-linearity in extension, Figure 2 shows a typical graph. The solid black line in this Figure corresponds to the linear limit of viscoelastic behavior and the colored lines show deviation of the linearity, higher rates correspond to lower deviation times [5,8].

Under no circumstance can the ratio be considered as some “apparent elongation viscosity” since no point in this graph corresponds to a steady flow (which is an obligatory condition in the definition of “viscosity”) and moreover, it may generally correspond to a flow-to-elastic deformation transition. This mistakable approach sometimes appears in discussions of experimental data.

Some recent reviews on the rheology of extension were published [9,10,11]. However, this line of research was outlined very quickly and the new experimental data and theoretical arguments have already accumulated and require analysis. This was conducted in a review based mainly on the publications of the last 5–7 years. 

Peculiarities of extension of polymeric liquids have rather significant value when we consider either melts or dilute (or semi-dilute) solutions. This is due to the different technological applications of these two groups of liquids. In the first case, we meet with the processing of polymers mainly by molding or extrusion. In the second case, we deal mainly with fiber spinning. Blow molding of films occupies the intermediate position. Therefore, it seems reasonable to separate this review into two parts devoted to melts and solutions. 

## 2. The Role of Elasticity in Polymer Processing

Any deformation in the polymer processing inevitably leads to molecular orientation that can be treated in terms of elasticity (stored energy) wherein normal stresses (under the extension flow) create a much higher effect than shear stresses (under the shear flow) that is clearly demonstrated in the simplest model of the deformation of a liquid drop inside a surrounding liquid under different modes of the flow [12,13,14]. Elastic (recoverable) force for a liquid droplet is surface tension while for polymers and in particular for polymer blends, the source of elasticity is molecular motion directed to the recovery of the equilibrium conformation and having the statistic (entropic) nature. Therefore, the inherent link between elasticity and elongation flow of viscoelastic polymeric liquids (solutions or melts) exists. 

In this section, we will consider the effects due to the elasticity of polymeric liquids, which are observed when flowing through capillaries (channels). In this regard, the original study [15] complements the presented review.

The role of elasticity associated with the technological practice of spinning fibers and blow molding is of independent interest. This is a separate topic that is beyond the scope of this review. Some individual aspects of this problem are considered in [16,17]. Molecular understanding of this issue will be considered in the next part of this paper.

**Figure 3:**
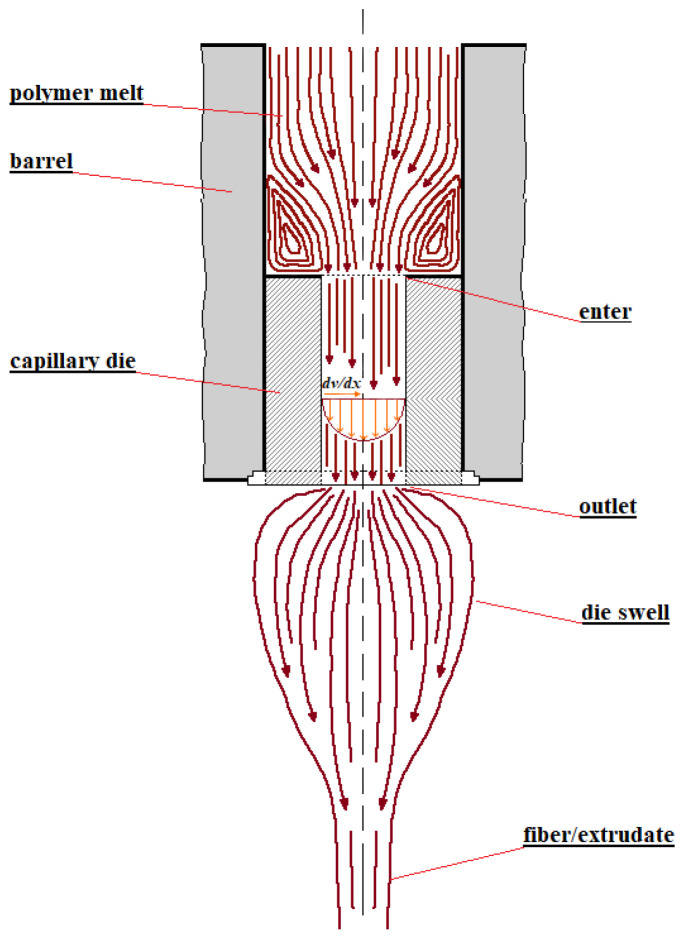
The velocity distribution in the flow through a cylinder die (capillary).

The role of extension is the most pronounced and important in shear flows through channels with variable cross-sections due to an obligatory change in the elongation velocity (i.e., the emergence of the rate of deformation). An evident example of such a case is extrusion. Figure 3 shows how velocity changes in the flow from a barrel with polymer melt, through a forming die (perhaps simply through a capillary), and in post-extrusion operations. There are two zones: the entrance into the die and the exit from it where elongation flow takes place.

**Figure 4:**
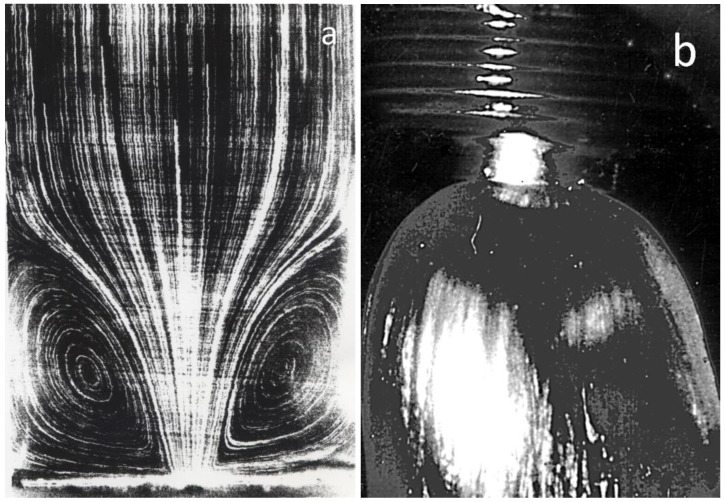
Typical effects associated with the elasticity of polymer melts: secondary flows at the entrance to a die (**a**) [18] and die swell after exit from a die (**b**) (authors’ photo).

As was said above, large deformations in the elongation flow of viscoelastic polymeric liquids relate to their elasticity, which manifests itself in the transition flows taking place at the entrance and exit zones. Figure 4 illustrates two characteristic effects related to the flow of polymeric liquids in these zones: the emergence of secondary flow (Figure 4a) and die swelling (Figure 4b) [18]. The quantitative manifestations of these effects depend on the deformation conditions and the nature of the polymer (see, for example, [19,20]

**Figure 5:**
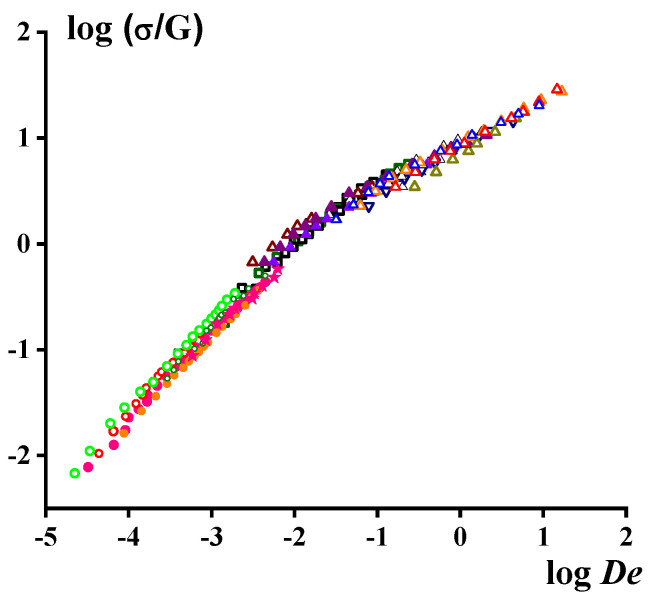
The reduced dependence of the shear stress on the Deborah number for different polymer melts and solutions—PAN solutions in DMSO, LDPE, polybutadiene, SAN, solutions of PIB in toluene—presented by different symbols (according to [21]).

The role of these two zones becomes dominant for short dies, since it is in these cases that the main part of losses (energy dissipation) happens due to transient (viscoelastic) regimes of the flow associated with extension. This suggestion was confirmed by the possibility of considering the dimensional pressure losses as a universal function of the Deborah number, De [21], where De is defined as the ratio of characteristic time of the segmental movement of macromolecules to the residence time in the channel. The generalized result of this approach is shown in Figure 5. Here, the shear stress is reduced by the plateau modulus *G* in the rubbery, *R*, zone and *De* is determined via the relaxation time in the flow, *F* (terminal), zone (as in Figure 1a). The existence of a common dependence on the shear stress on the characteristic relaxation time (built in the reduced coordinates) indicates the decisive role of elasticity in the flow through short dies.

Secondary flows are a phenomenon first examined by L. Prandtl (1926) for Newtonian liquids, which are characterized in terms of the cross-plane component of the mean kinetic energy. Nowadays this effect is primarily considered by numerical methods [22,23]. This problem has received the new content for viscoelastic fluids, in which the Reynolds number is not the determining factor, and the elasticity of the liquid is expressed by the Weissenberg number [24,25,26]. Secondary flows in elastic liquids appear at very low Reynolds numbers and are associated with elastic turbulence. Their quantitative description depends on the choice of the rheological model due to the different approaches for the characterization of elastic non-linearity, and solutions of dynamic problems (such as in a simpler case of Newtonian liquids) are reached using numerical analysis methods. In the limits of this review, it is essential that secondary flows always increase the hydrodynamic resistance [27].

In rheological measurements and in technological practice, the total entrance phenomena (including secondary flows and extension) are usually characterized by an end correction, *n*, as a measure of some conventional increase in the length of the die. Then, the true shear stress, *σ*, at the wall of the capillary is expressed as

(1) $$\sigma =\frac{\Delta PR}{2\left(L+nR\right)}=\frac{\Delta P}{2\left(L/R+n\right)}$$

where Δ*P* is the difference in pressure at the entrance and exit of a capillary and *R* and *L* are the radius and the length of a capillary, respectively. 

Sometimes, the gradient of the elongation velocity is used for calculating a conditional “elongation viscosity” $\eta^{+}=\sigma_{E}/\dot{\varepsilon }$, although (as explained above) this value cannot be treated as “viscosity”. Based on experimental evidence, it was shown that this value does not have any reasonable meaning for the capillary flows of viscoelastic polymer melts [28].

**Figure 6:**
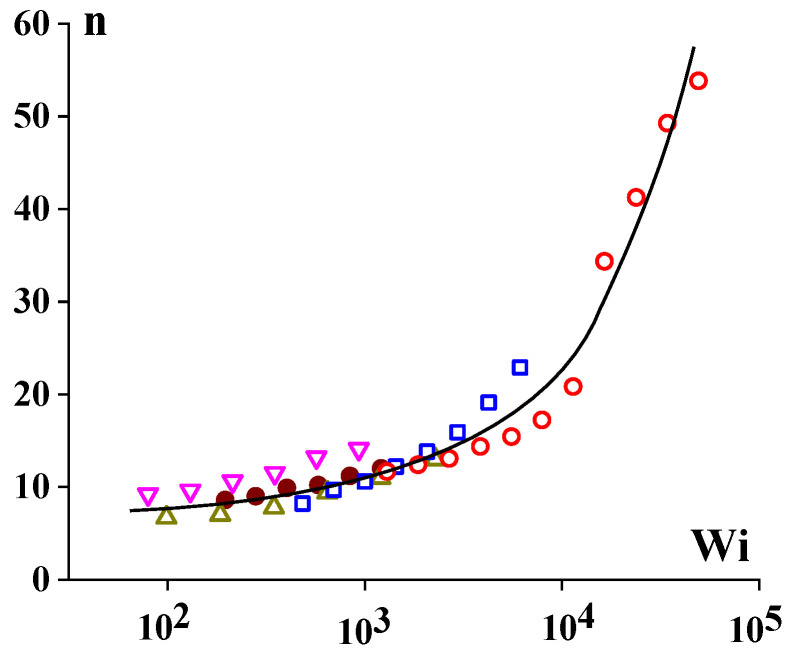
Dependence of end correction on the Weissenberg number. Different symbols correspond to various compositions of low- and high- molecular-weight polyethylenes (according to [28]).

The end correction (summing all entrance additional energy losses) depends on the viscoelastic properties, and its relative contribution to the total pressure loss is determined by the ratio between *L/R* and *n*, i.e., the capillary length (as seen from Equation (1)). In this sense, in addition to the experimental data presented in Figure 5, the *n*(Wi) dependence is shown in Figure 6.

The physics behind the increase in end correction alongside an increase in the *Wi* number is most likely related to the increase in elastic deformations depending on shear stress (or shear rate), but not with hydrodynamic reasons as in Cogsell’s model. This occurs simultaneously with the development of the non-Newtonian effect. It is then reasonable that elasticity correlates with the degree of non-Newtonian effect. Indeed, it was found that the ratio of the apparent viscosity, $\eta \left(\dot{\gamma }\right)$, to the initial (maximal) Newtonian viscosity, $\eta_{0}$, can be considered as a function of the stored elastic energy, *W*, i.e., the elasticity of the liquid [29] and expressed as

(2) $$\frac{\eta \left(\dot{\gamma }\right)}{\eta_{0}}=e^{\beta W/RT}$$

where *R* is the universal gas constant, *T* is absolute temperature, and *β* is an individual constant of an elastic liquid. The stored elastic energy is calculated as

(3) $$W\left(\dot{\gamma }\right)=\int_{0}^{\gamma_{el}}\sigma \left(\dot{\gamma }\right)d\gamma_{el}$$

Here, $\sigma \left(\dot{\gamma }\right)$ is the dependence of the shear stress, *σ*, on the shear rate, $\dot{\gamma }$, in a stationary flow (i.e., the flow curve) and $\gamma_{el}=\gamma_{el}(\dot{\gamma })$ is the elastic deformation at this shear rate. Independent measurements of the values in the right and left sides of Equation (2) for many different polymeric liquids confirmed the correction of this relationship.

**Figure 7:**
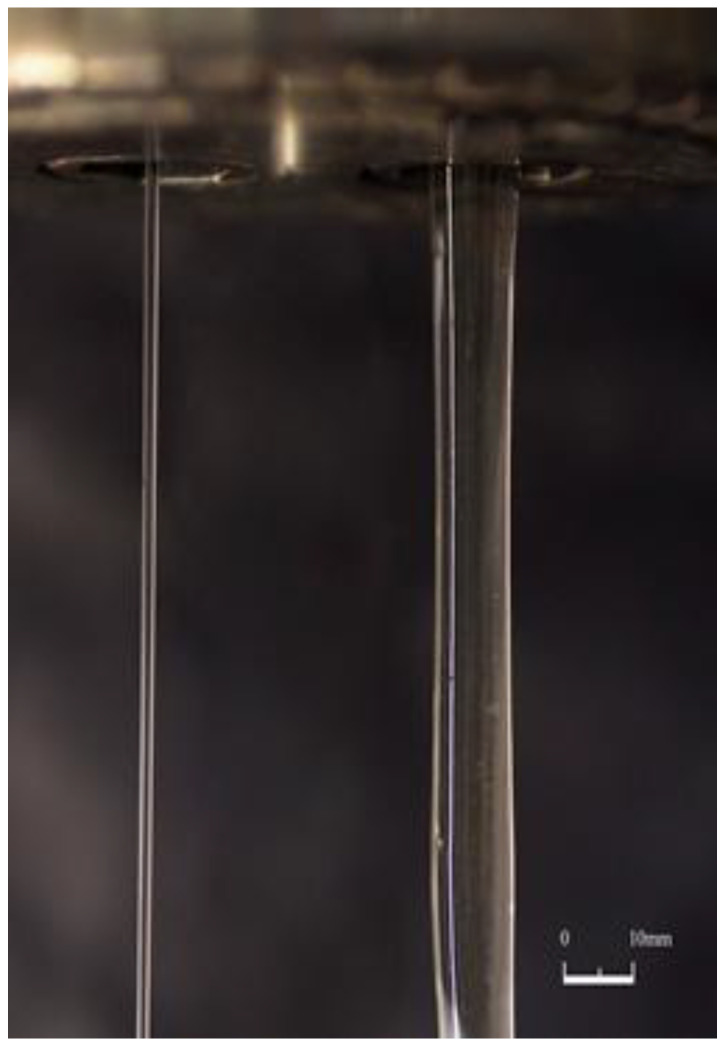
Comparison of two jects obtained at the same volume output but with different lengths of capillaries, long (**left**) and short (**right**), authors’ photo.

Elastic deformations stored at the inlet due to the convergent flow relax after leaving the capillary and lead to the die swelling (Figure 4b). This phenomenon depends on the capillary length since the additional stresses at least partially relax when passing through the capillary, and thus the jet diameter depends on the value of *L/R*. This is illustrated by the photo (Figure 7) wherein two jets obtained at the same given volume output are shown but with long (left) and short (right) dies [28].

The physics behind the die swell is rather evident. This is because of the release of stored elastic energy due to the recoverable conformation of the entangled polymeric chains. As said above, the quantitative measure of this effect depends on the length of the die (capillary). The die swell should be taken into account in the design of the processing equipment [30], and this is especially important when designing spinnerets in fiber spinning since the dies in these devices are always very short. 

The mechanics of die swell was considered in many publications by numerical methods based primarily on the analysis of rather complex rheological equations [31,32]. This means that the theoretical base for such calculations is ready. However, its practical application requires knowledge of a large amount of information about the rheological properties of the processed polymer. Then, it is reasonable to apply the theoretical models in large-scale industrial production. Therefore, the problem of measuring die swell continues and many authors try to carry out the direct measurement for certain applications in extrusion polymer profiles [33,34]. A new aspect of this problem is associated with the extrusion of filaments for 3D printing (additive technology) [35,36].

**Figure 8:**
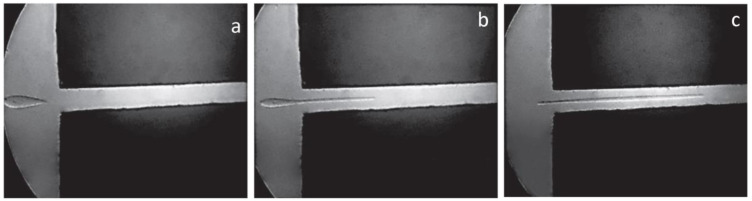
Successive stages of the deformation of a drop during the transition from a wide volume to a narrow capillary. Initial (**a**), intermediate (**b**), and final (**c**) stages of the process (reproduced from [37] with permission.

The effect of extension (longitudinal deformations) at the capillary entrance becomes rather evident when we observe the flow of two liquids forming emulsions. This takes place in the flow of blends of immiscible polymer melts. The results of a model experiment are shown in Figure 8, where the deformation of a liquid droplet during the transition from a wide to narrow channel is illustrated [37].

**Figure 9:**
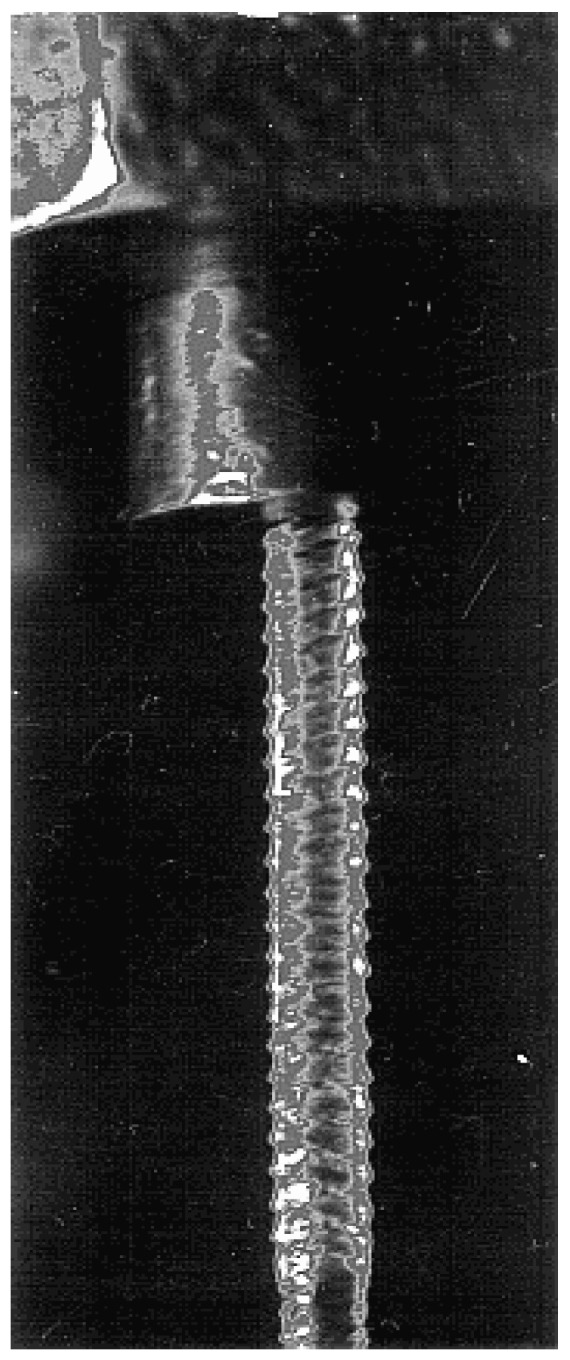
Self-oscillations at the exit of a capillary.

The effect of self-oscillation during the high-speed extrusion of polymer melts (Figure 9) is a well-known and well-documented phenomenon [38]. Its physics, associated with the elastic rupture at the point of singularity on the exit section of a capillary, was qualitatively described by the old Cogswell model [39]. Self-oscillations represent the initial stage of instability in the flow of viscoelastic fluids. A detailed consideration of this issue is presented in the review [40].

**Figure 10:**
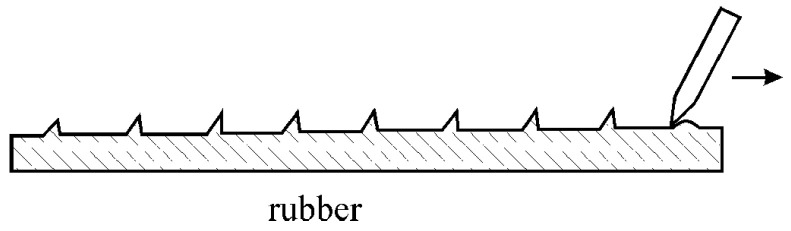
Self-oscillations due to the movement of a scribe along the rubbery surface.

This effect can be understood as an analog of the spurt effect. At high shear stresses, melt becomes elastic, and the edge of the capillary exit plays a role of a scrapper sliding along the rubbery surface (Figure 10). This is a reverse picture of the movement of a rubbery-like melt at the edge of the capillary. Although the appearance of periodic surface self-oscillating defects on the surface of extrudates (also known as the shark skin) associated with elastic ruptures at a singular point was described in many publications, its quantitative theory is still absent.

The next case of the influence of elasticity on the shear flow is associated with the interaction of a polymer fluid with the wall. This is a well-known effect of spurt associated with wall slip. This is also the case with using a rather mild measuring system in rotary rheometers [41]. A rigorous solution of this problem would be also interesting. 

We can also observe the consequences of elastic deformations of polymer melts preserving frozen residual stresses and shape memory in articles obtained by molding or extrusion. It is quite evident that residual stresses and memory effects are associated with orientation depending on the stress in shear flow (see, e.g., Figure 25 in [42]) and related to its elasticity. One can find a description of frozen stresses, their direct observations, and the influence of this phenomenon on the performance of final products in numerous publications (e.g., [43,44,45]) and there is no doubt in its practical importance [46]. However, the general theoretical model of this phenomenon is absent although some attempts for stimulating calculations for frozen stresses are known [47]. One of the earlier attempts to solve this problem, by constructing a rigorous system of equations, clearly demonstrated the correct way to do this as well as numerous difficulties encountered [48]. Indeed, the practical application of thermoviscoelastic problems requires a large amount of experimental information about the temperature dependences of rather complicated rheological properties of the material, as well as overcoming computational difficulties in solving a system of non-linear or integral equations. Nevertheless, we can be sure that this problem will attract the attention of professionals due to its practical importance in the processing and application of engineering plastics. 

**Figure 11:**
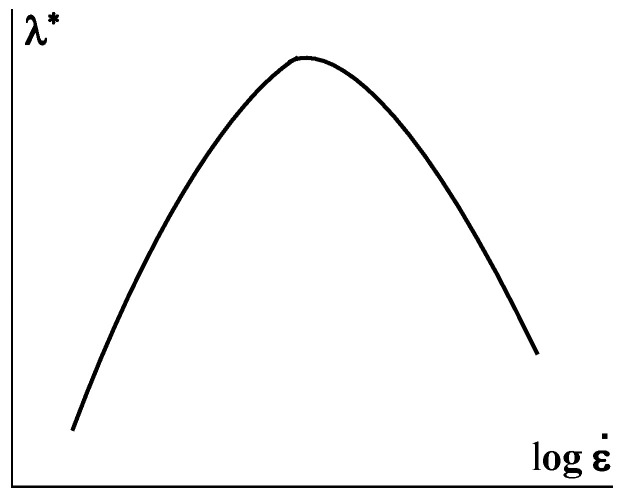
Correlation between the draw rate and the ultimate strain.

Although the elasticity of polymer liquids is their inherent property and is important at all stages of the traditional processing, it is extension that is the mode of deformation, where elasticity plays a decisive role due to direct correlation with the orientation of macromolecules and the influence of this factor on the technical properties of spun fibers. In fact, the extension of fibers in different stages of the technological process occurs due to the uncoiling of macromolecules associated with elastic deformations. Then, the increase in ultimate strain (at break) λ*, as well as the strength of a matter, correlates with the draw rate. This should be an elastic drawing and a further increase in the draw rate can lead to the deformation-induced glass transition with a decrease in λ*. This is shown in Figure 11, and the shape of the curve is obviously similar to the right part of the envelope curve in Figure 2.

The universal modeling of extension presented in Figure 2 is in accordance with the generalized model [6] and is valid for homogeneous stretching. The extension of polymeric liquids in the elastic domain of deformations can occur with neck formation similarly to necking in solid polymers and further stretching entails theelastic yielding with the transition of homogeneous filament to the neck. This yielding effect happens at critical strain and finally results in the elastic breakup at non-uniform extension [49]. The concept of necking under extension was analyzed by stability analysis of the stretching process, which made it possible to obtain criteria of this effect corresponding to the existing phenomenological models of a non-linear viscoelastic liquid [50]. In some publications, the appearance of a plateau in the stress vs. deformation curve near the breakup point was described. This plateau takes place for linear polymers [51] as well as for ring macromolecules with an unusual sharp increase in the apparent viscosity [52]. The nature of this effect is not evident. The authors of original publications treat it as flow, although possibly this yielding happens due to the necking phenomenon.

Modeling the polymeric liquid bridges leading to failure during extension was discussed in [53]. However, it is necessary to keep in mind that the mechanism of fracture of polymeric liquids under tension depends on the draw rate, since the latter is determined by the relaxation state of the polymer and in particular the liquid flow-to-elastic state transition.

At rather low draw ratios, the classical Rayleigh–Plateau breakup of liquid jets due to surface tension disturbances is observed [54]. In the rubber-like state (in the medium range of draw rate), the breakup occurs quite in the same mode as for usual rubbers. However, at high deformation rates, the breakup is initiated by the appearance of the simultaneous propagation of multiple cracks while their position is random, similarly to how it happens in the rupture of different solids [55,56].

A rather different understanding of the mechanism of rupture in extension was proposed in [57,58]. The authors assume that there are only two different states associated with the regimes of deformations: the liquid and elastic solid. They proposed the cohesive failure model based on the entropic fracture hypothesis. According to this model, the rupture of the bond in the main macromolecular chain was assumed as the basic mechanism of the brittle breakup of a filament. This approach was criticized in [59], where contrary to the hypothesis that chains are fully uncoiled and scission in melt rupture is due to an “entropic fracture” mechanism, it was declared that sufficient enthalpic changes associated with conformational distortions at the bond level take place. 

## 3. Elasticity in the Dynamics of Extension of Polymer Solutions

### 3.1. General Equations

**Figure 12:**
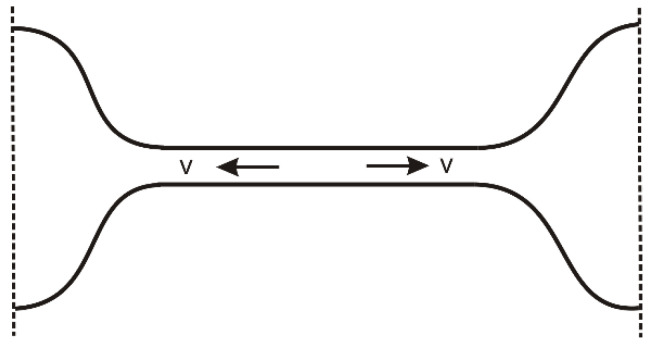
Schematic picture of a thread (bridge) connecting two droplets.

**Figure 13:**
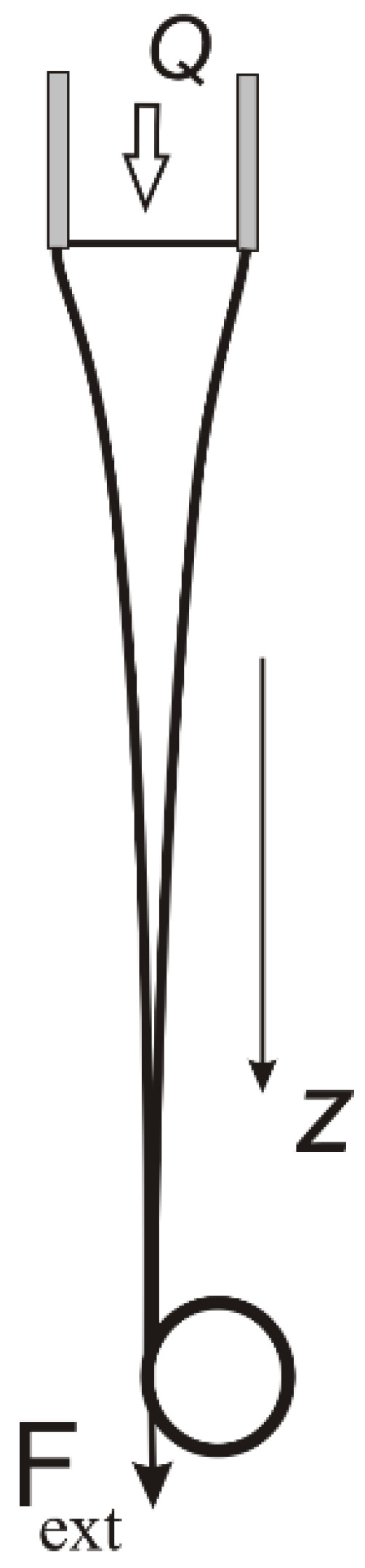
Stretching of a polymer solution jet by an external force $F_{ext}$ during continuous fiber spinning.

In the previous part, we considered the features of the elastic behavior of polymeric liquids during technological processing, including both shear and extension. In recent years, significant progress has also been made in studying polymer solution behavior under extension. In this part, we mainly focus on elucidating the role of elasticity using the theoretical methods. We will consider two cases: a thread (bridge) connecting two droplets and self-thinning under the action of capillary forces (Figure 12), and a stationary jet stretched under the action of an external force after the solution leaves the orifice with a fixed flow rate. The external force can be mechanical and applied to the free end of the jet, as in the case of fiber drawing (Figure 13), as well as gravitational or electrical (electrospinning). In the latter case, the force is applied to the entire jet.

First, let us formulate the basic equation for the balance of forces in the volume of liquid in the general case, including the inertial, viscoelastic, gravitational, and electrostatic forces. Denoting the density of the solution as ρ and assuming that the electric field inside the fluid is $E_{i}\left(x,t\right)$ and the velocity is v(x,t), the momentum equation is written as [54,60,61,62]

(4) $$\rho \frac{\partial v}{\partial t}+\rho v\cdot \nabla v-\nabla \cdot \left(\Sigma -pI\right)-\rho g-qE_{i}=0$$

where the velocity v(x,t) obeys the incompressibility condition ∇⋅v=0. Here $x=(x_{1},x_{2},x_{3})$ is the coordinate, *t* is the time, p is the pressure, ∇ is the gradient operator, Σ is the stress tensor, ρg is the gravity force density, $qE_{i}$ is the electric force density, and ($q=e\left(n_{+}-n_{-}\right)$ is the free charge density, where $n_{+}$ and $n_{-}$ are concentrations of positively and negatively charged monovalent ions having the charge *e* respectively) and I is the unit tensor. The differential Equation (4) should be supplemented by the boundary condition on the free surface. This condition implies the balance of the viscoelastic, capillary, and electric forces.

(5) $$p_{s}n-\Sigma \cdot n-\gamma Cn+F=0$$

Here, $p_{s}$ is the pressure at the surface, n is a normal vector to the surface, C=divn is the total surface curvature, and γ is the surface tension. The electric force F acting per unit area is given by:

(6) $$F=\varepsilon_{0}\left(E_{o,n}E_{o}-\varepsilon E_{i,n}E_{i}\right)-\frac{\varepsilon_{0}}{2}\left(E_{o}^{2}-\varepsilon E_{i}^{2}\right)n$$

where $E_{o}$ is the electric fields outside the liquid. The electric fields $E_{i}$ and $E_{o}$ are found from the electrostatic equations [60,61,62,63].

To study the dynamics of the rectilinear jet (thread), a cylindrical system of coordinates will be used. Assuming that the jet surface is described by an axisymmetric function a=a(z,t), the normal (n) and tangential (τ) vectors to the surface are given by:

(7) $$n=-\frac{{a^{\prime }}_{z}}{\sqrt{1+{a^{\prime }}_{z}^{2}}}e_{z}+\frac{1}{\sqrt{1+{a^{\prime }}_{z}^{2}}}e_{r},\;\tau =\frac{1}{\sqrt{1+{a^{\prime }}_{z}^{2}}}e_{z}+\frac{{a^{\prime }}_{z}}{\sqrt{1+{a^{\prime }}_{z}^{2}}}e_{r}$$

Here, **e***_z_* and **e***_r_* are the unit vectors directed along and perpendicular to the jet axis, respectively. 

Analysis of the three-dimensional momentum, Equation (4), with the boundary conditions (5) is a very difficult mathematical problem. In the case of axisymmetric rectilinear jet, the problem can be simplified using a slender body approximation since the profile of the jet slowly changes along the extension axis *z*, $\left|{a^{\prime }}_{z}\right|\ll 1$ (${a^{\prime }}_{z}=\frac{\partial a}{\partial z}$). To derive the corresponding one-dimensional momentum equation, let us consider the jet section [z,z+dz] [64]. After multiplying the Equation (1) by the vector **e***_z_* and integration over this section, we obtain the equation for the velocity component $v_{z}^{}$:

(8) $$\int_{0}^{a(z,t)}rdr\left(\rho \frac{\partial v_{z}^{}}{\partial t}-\rho g-qE_{z}\right)+\frac{\partial }{\partial z}\left[\int_{0}^{a(z,t)}rdr\left(\rho v_{z}^{2}+p-\Sigma_{zz}\right)\right]+a\sqrt{1+{a^{\prime }}_{z}^{2}}\left(\rho vv+p_{s}I-\Sigma \right)\cdot ne_{z}=0$$

where the gravity acts along the z-axis. The incompressibility condition ∇⋅v=0 after integration over the section [z,z+dz] and subsequent use of the kinematic equation $\frac{\partial a}{\partial t}+v_{z}{a^{\prime }}_{z}-v_{r}=0$ reduces to the mass conservation equation:

(9) $$\frac{\partial a^{2}}{\partial t}+\frac{\partial }{\partial z}\left(a^{2}v_{z}^{}\right)=0$$

Elimination of the pressure from Equation (8) (note, within the framework of the slender body approximation $p≃p_{s}$) by taking into account the boundary condition (5) we arrive at the well-known form of the momentum equation:

(10) $$\frac{\partial }{\partial t}\left(\rho a^{2}v_{z}^{}\right)+a^{2}\frac{\partial }{\partial z}\left(\gamma C-F_{n}\right)+\frac{\partial }{\partial z}\left[a^{2}\left(\rho v_{z}^{2}+\Sigma_{nn}-\Sigma_{zz}\right)\right]≃2aF_{\tau }+qa^{2}E_{z}+\rho ga^{2}$$

where $F_{\tau }=F\cdot \tau$, $F_{n}=F\cdot n$ and $E_{z}=E\cdot e_{z}$. Furthermore, depending on the system under consideration, it is necessary to determine the stress tensor, and in the case of electrospinning, to add electrostatic and charge balance equations.

For the Newtonian liquid the stress tensor is $\Sigma =\eta \left(\nabla v+{\left(\nabla v\right)}^{T}\right)$ where η is the viscosity and

(11) $${\left(\nabla v\right)}_{ij}=\frac{\partial v_{j}}{\partial x_{i}},{\left(\nabla v\right)}_{ij}^{T}=\frac{\partial v_{i}}{\partial x_{j}},\;i,j=1,2,3$$

are the velocity gradients. When considering polymer solutions exhibiting viscoelastic behavior, additional equations are required to determine the stress tensor [64,65,66]. Two approaches are possible here: phenomenological and molecular. In the phenomenological approach, the stress tensor is determined by the constitutional equation. The Maxwell, Oldroyd B, and FENE-P equations are often used to describe extension of polymer solutions without entanglements. The polymer chains in these rheological models are described by elastic dumbbells with constant friction. The most general is the FENE-P model. It captures the viscoelastic effects, as well as those in strong elongational flows when the finite extensibility of the polymer chains is important [64,65]. The polymer chain is modeled by a non-Hookean dumbbell with the extension force $f=\frac{3k_{B}T}{R_{0}^{2}}\frac{R}{1-R^{2}/L^{2}}$ which is related to the elastic energy $F_{el}=-\frac{3k_{B}TL^{2}}{2R_{0}^{2}}\ln\left(1-\frac{R^{2}}{L^{2}}\right)$. Here, $k_{B}$ is the Boltzmann constant, T is the temperature, R is the distance between the beads, L is the maximum spring length, and $R_{0}^{2}\propto L$ is the mean-square equilibrium distance between the beads. 

The FENE-P model equations are formulated in terms of a conformation tensor A=〈RR〉 where the angular brackets denote averaging over the distribution of the vector R. The stress tensor Σ is a sum of the solvent stress $\Sigma_{s}=\eta_{s}\left(\nabla v+{\left(\nabla v\right)}^{T}\right)$ where $\eta_{s}$ is the solvent viscosity and the polymer stress $\Sigma_{p}$:

(12) $$\Sigma =\Sigma_{s}+\Sigma_{p},\;\Sigma_{p}=G\frac{A/R_{0}^{2}-I}{1-\mathrm{tr}A/L^{2}}$$

with A obeying

(13) $$\tau \left[\frac{\partial A}{\partial t}+\left(v\cdot \nabla \right)A-{\left(\nabla v\right)}^{T}\cdot A-A\cdot \nabla v\right]+\frac{A-R_{0}^{2}I}{1-\mathrm{tr}A/L^{2}}=0$$

Here, the elastic modulus is $G=3nk_{B}T$ where is the concentration of polymer chains (springs) and τ is the relaxation time. The linear viscosity of the polymer component is expressed by means of the scaling relation $\eta_{p}≃G\tau$. The Oldroyd B model assumes infinite extensible polymer chains (L→∞) and the Maxwell model also does not take into account the solvent ($\Sigma_{s}=0$).

### 3.2. Capillary Thinning of a Polymer Solution Thread

One of the important and most studied systems is the liquid bridge connecting two droplets, Figure 12. The bridge can form, for example, after the separation of two planes containing liquid in the gap. Then, it becomes thinner due to the action of capillary forces. The breakup dynamics of a Newtonian liquid bridge (the normal stress difference in this case is $\Sigma_{zz}-\Sigma_{rr}=3\eta \frac{\partial v_{z}}{\partial z}$) is related to its Ohnesorge number $\mathrm{Oh}=\eta /\sqrt{\rho \gamma a}$. If the bridge is thick enough (Oh≪1), the inertial and capillary forces dominate, and the inertia–capillary regime or IC regime is realized. In this case, the minimum thread radius (the radius of the neck) obeys the scaling law $a_{\min}(t)=A{\left(\gamma /\rho \right)}^{1/3}{\left(t_{b}-t\right)}^{2/3}$ [66,67], where $t_{b}$ is the putative breakup time. Different values for the prefactor *A* were proposed and used: *A* ≈ 0.4 [68], *A* ≈ 0.64 [69], and *A* ≈ 0.717 [70]. The characteristic breakup time of the thread is $\tau_{I}≃2.9\sqrt{\rho a^{3}/\gamma }$ [71,72] and the local Reynolds number is large in this regime: Re∼1/Oh≫1. At high Ohnesorge numbers, Oh≫1, another visco-capillary regime, or VC regime, arises. It occurs in highly viscous liquids or in relatively thin threads. Inertial effects are negligible in this regime: Re≪1, and the breakup time is $\tau_{V}=6\eta a/\gamma$ [67,73]. The neck radius decreases linearly in time, $a(t)=0.07\left(\gamma /\eta \right)\left(t_{b}-t\right)$ [74,75]. The Ohnesorge number reflects the ratio of two timescales, $\tau_{V}$ and $\tau_{I}$: $\mathrm{Oh}∼\tau_{V}/\tau_{I}$. Both regimes fail close to the breakup point, and a new visco-inertial regime emerges wherein both the inertia and the viscosity are equally important while the local Reynolds number is close to one [73]. 

The break-up of a polymer solution proceeds in a much more complicated way due to viscoelasticity. Early experimental [76,77,78] and theoretical [79,80] studies have revealed an important role of elasticity associated with the transition of polymer chains to an elongated state. The addition of high-molecular weight polymers in a low-viscosity solvent leads to the formation of long-lived bridges between the droplets even at very low polymer concentrations [81,82]. The dynamics of the bridges are described by two additional modes associated with the elasticity and finite extensibility of polymer chains [83,84,85]. The elasto-capillary (EC) regime is associated with the unfolding of polymer coils and the predominance of viscoelastic and capillary forces. The terminal quasi-Newtonian visco-capillary (TVC) regime is characterized by the almost complete orientation of macromolecules along the stretching axis [68,83,84]. Unfolding of polymer coils can already start in the IC regime. Both in the IC and VC regimes, the rate of stretching of the thread increases according to the law $\dot{\varepsilon }(t)=-\frac{2\dot{a}}{a}\propto {\left(t_{b}-t\right)}^{-1}$. This leads to an increase in the Weissenberg number $Wi=\dot{\varepsilon }\tau$ where τ is the characteristic relaxation time of the quiescent polymer solution. The transition to the EC regime occurs at Wi ~ 1. The EC regime was widely studied theoretically using the force balance equations, and the viscoelasticity of the polymer solutions was taken into account mainly on the basis of the classical constitutive equations of the Oldroyd-B and FENE-P models [79,80,85,86,87,88,89,90]. According to these theories, the radius of the thread *a* in the EC regime decreases as $a(t)\propto e^{-t/3\tau }$. The exponential law was observed in many experiments with dilute, semi-dilute, and concentrated polymer solutions using CaBER, DoS, and ROJER rheometry including visualization of the thinning dynamics [67,69,91,92,93,94,95,96]. 

The dynamics of the bridge in the EC regime can be described by Equation (7) after elimination the gravity and electrostatic forces. Assuming that the curvature C≃1/a, Formula (6) obtains

(14) $$\rho \frac{\partial v_{z}^{}}{\partial t}+\rho v_{z}^{}\frac{\partial v_{z}^{}}{\partial z}=\frac{1}{\pi a^{2}}\frac{\partial }{\partial z}\left(\pi \gamma a+\pi a^{2}\left(\Sigma_{zz}-\Sigma_{rr}\right)\right)$$

This equation should be supplemented with appropriate boundary conditions in the transition region from the thread to the droplet. These conditions are determined through the thread tensile force, which is the sum of the surface and body forces: $T=2\pi \gamma a+\pi a^{2}\left(\Sigma_{zz}-p\right)$ [88]. This force generally differs from the net capillary force 2πγa and can be written as [M2] T=2πγaX where *X* depends on the ratio $\Sigma_{zz}/p$ [96,97]. The pressure is found from the boundary condition $p=\gamma /a+\Sigma_{rr}$, hence

(15) $$\gamma /a+\Sigma_{zz}-\Sigma_{rr}=T/(\pi a^{2})$$

In the EC regime, the radius of the thread is nearly constant along the axis, i.e., a≃a(t) and the axial stress obey inequalities $GN\gg \Sigma_{zz}^{p}\gg G$ and $\Sigma_{zz}^{p}\gg \Sigma_{rr}^{p}$, at that contribution from the solvent, can be omitted: $\Sigma_{zz}-\Sigma_{rr}≃\Sigma_{zz}^{p}≃G\left(R_{z}^{2}/R_{0}^{2}\right)$. Therefore, Equations (9) and (10) are simplified in the EC regime:

(16) $$\tau \frac{d}{dt}\Sigma_{zz}^{p}-2\dot{\varepsilon }\tau \Sigma_{zz}^{p}+\Sigma_{zz}^{p}=0$$

In Equation (16) $\frac{d\Sigma_{zz}^{p}}{dt}=\frac{\partial \Sigma_{zz}^{p}}{\partial t}{+v}_{z}\frac{d\Sigma_{zz}^{p}}{dz}$ and $\dot{\varepsilon }=-\frac{2}{a}\frac{\partial a}{\partial t}$. Based on the use of various theoretical methods, it is shown that the thread tension force in the EC mode is equal to T=3πγa, and the force balance equation in EC regime is written as $\Sigma_{zz}-\Sigma_{rr}≃\Sigma_{zz}^{p}≃2\gamma /a$ [88,97,98]. The evolution of the thread radius is found from Equation (16). It changes over time as $a(t)=a_{0}{\left(\frac{\Sigma_{0}a_{0}}{2\gamma }\right)}^{1/3}e^{-t/3\tau }$ where $a_{0}=a\left(0\right)$ is the initial radius and $\Sigma_{0}$ is the initial stress, $\Sigma_{0}\geq G$ [88]. The experimental measurements of the stresses acting in the capillary bridge connecting the droplets were performed by Bazilevskii et al. [99,100,101]. 

The Weissenberg number in the EC regime is constant, $Wi_{EC}=\dot{\varepsilon }\tau =2/3$, whereas in the IC regime it increases in time as $Wi_{IC}=\frac{4\tau }{3\left(t_{b}-t\right)}$, and in VC regime as $Wi_{VC}=\frac{\tau }{\left(t_{b}-t\right)}$. The transition from the VC to EC regime is associated with the beginning of coil unfolding, whereas the transition from IC to EC is determined by the change in the balance of forces. The unfolding of chains in the latter case begins already in the IC mode. The value of the Weissenberg number at the transition point is estimated from the condition that the viscoelastic force becomes the order of the capillary force, i.e., $\Sigma_{zz}≃2\gamma /a$ where the stress component $\Sigma_{zz}$ is found from Equation (13) with $\dot{\varepsilon }=\frac{4}{3\left(t_{b}-t\right)}$: $\Sigma_{zz}≃G{\left(\frac{8\tau }{3(t_{b}-t)}\right)}^{8/3}$ where $t_{b}-t=A^{-3/2}{\left(\rho a^{3}/\gamma \right)}^{1/2}$. The Weissenberg number at the transition point follows from the force balance equation: $Wi^{*}∼{\left(\frac{\gamma^{2}\rho \tau }{\eta_{p}^{3}}\right)}^{1/6}$. After the IC to EC transition point, the Weissenberg number must decrease to the value $Wi_{EC}=2/3$, i.e., *Wi* first increases in the inertial regime as $Wi\propto {\left(t_{b}-t\right)}^{-1}$, and after passing through the maximum it decreases. The non-monotonic behavior of $\dot{\varepsilon }$ with time was observed in ref [69]. 

When the macromolecules become almost fully elongated ($A_{zz}≃L^{2}$), the EC regime transformed to the TVC regime with $\Sigma_{p}≃\Sigma_{zz}^{p}≃2\eta_{p}(L^{2}/R_{0}^{2})\dot{\varepsilon }$ and the radius of the thread decreases linearly in time, $a(t)∼(\gamma /\eta_{eff})\left(t_{b}-t\right)$ [68]. The effective viscosity $\eta_{eff}$ in the TVC regime is $\eta_{eff}∼\eta_{p}N$.

Experiments show that the apparent relaxation time τ coming from fitting a(t) in the EC regime significantly increases with a concentration in the dilute solution regime ($c\ll c^{*}$ or $\phi \ll \phi^{*}$ where ϕ is the volume fraction of polymer) [102]. These results are at odds with the Rouse–Zimm theory for dilute solutions, in which the relaxation time depends on the molecular weight, and the concentration dependence due to hydrodynamic interactions between the chains is weak [103]. This contradiction triggered questions on how to define a dilute solution and how interchain interactions affect the rheology of solutions in extensional flow [104,105,106]. 

The effect of hydrodynamic interactions on the thread dynamics can be taken into account using the molecular approach. One such approach in the case of a semi-flexible chain solution was formulated in the ref. [107]. The relaxation of the semi-flexible chain of contour length L, diameter *d,* and the Kuhn segment length l (d≪l≪L) in dilute solutions in the presence of a flow can be described by the equation on the orientational (stretching) parameter $s=R_{z}/L$ where $R_{z}$ is the end-to-end distance of the chain [107], taking into account the hydrodynamic interactions:

(17) $$\tau_{R}{\left(1-s^{2}\right)}^{2}\left(\frac{ds}{dt}-\dot{\varepsilon }s\right)=-1-\frac{1}{3}\left(s^{4}-2s^{2}\right)$$

where $\tau_{R}=\frac{\pi }{18}\frac{\eta_{s}lL^{2}}{k_{H}T}$ is the Rouse relaxation time. At equilibrium, the orientational parameter is $s_{0}≃R_{0}/L=\sqrt{l/L}\ll 1$. According to Equation (17), polymer coils begin to unfold if the condition $\tau_{Z}\dot{\varepsilon }>1$ is satisfied where $\tau_{Z}=\tau_{R}s_{0}∼\frac{\pi }{18}\frac{\eta_{s}}{T}R_{0}^{3}$ is the Zimm relaxation time. Notably, the elasticity of a semi-flexible chain is approximately described by a non-Hookean dumbbell with elastic energy $F_{el}≃\frac{3k_{B}TR^{2}}{2R_{0}^{2}}\frac{\left(1-R^{2}/3L^{2}\right)}{1-R^{2}/L^{2}}$. 

In the EC regime, the polymer part of the axial stress $\Sigma_{zz}^{p}$ exceeds the radial component $\Sigma_{rr}^{p}$, $\Sigma_{rr}^{p}\ll \Sigma_{zz}^{p}$, and 1−s≪1, therefore, the normal stress difference is $\Sigma_{p}^{}\equiv \Sigma_{zz}^{p}-\Sigma_{rr}^{p}≃\frac{3ck_{B}T}{N}\frac{R_{z}^{2}}{R_{0}^{2}}$. Notably, this expression is similar to that in the Oldroyd B model with $A_{zz}=R_{z}^{2}$ since $R_{z}\gg R_{0}$. The radius of the thread a(t) and the axial end-to-end distance $R_{z}(t)$ in the EC regime are found in Equation (17) after taking into account the force balance equation $\Sigma_{p}∼2\gamma /a$:

(18) $$R_{z}∼\frac{L}{3}\left(t/\tau_{R}\right),\;a∼a_{1}{\left(\tau_{R}/t\right)}^{2},\;\dot{\varepsilon }≃4/t$$

Here, $t\leq \tau_{R}$ and $a_{1}=\frac{3\pi }{4}\frac{\gamma ld^{2}}{\phi T}$ [107]. The power law $a\propto t^{-2}$ arises due to a linear dependence of the friction force on the longitudinal size of the chain that is a consequence of hydrodynamic interactions. It should prevail for dilute polymer solutions with concentration $c\ll c^{*}$. The rate of extension, $\dot{\varepsilon }(t)$, in the thinning process shows a non-monotonic time-dependence: it first increases as $\dot{\varepsilon }=(4/3)/(t_{b}-t)$ in the inertial regime but then decreases as $\dot{\varepsilon }≃4/t$ in the viscoelastic regime. 

To explain the discrepancy between the above theory and experiments with dilute solutions in the EC mode, which show an exponential thinning of the thread, the formation of transition bonds between monomers of different chains upon their contact was proposed [107]. Such bonds can exist, for example, in aqueous solutions of PAM [83,84] or PEO [108,109,110]. If the lifetime $\tau_{b}$ of a bond is long, $\tau_{b}\gg \eta_{s}d^{3}/T$, the polymer chain dynamics become Rouse-like with a high effective friction per chain which is proportional to the bond lifetime and the number of bonds $n_{b}∼\phi N$, i.e., it is proportional to the number of monomers. Therefore, the chain relaxation time is $\tau_{R}^{*}∼\tau_{b}\phi^{2}N^{2}\gg \tau_{R}$ and the chain dynamics should be the Rouse type [107,111]. The increase in the relaxation time $\tau_{R}^{*}$ with the polymer concentration is in qualitative agreement with the experiment. However, experimentally, a weaker dependence is observed [68,69,85,91,102,104]. 

### 3.3. Blistering Instability

**Figure 14:**
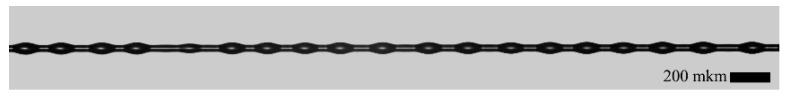
Blistering structure formed by continuous drawing out of a 25% solution of PAN (M_w_ = 85,000) in DMSO. Authors’ photo.

One of the interesting phenomena observed in polymer threads is the appearance of pearling or blistering structures at the end of the exponential thinning regime when the polymer chains are highly stretched [78,112,113,114,115,116,117,118,119,120], Figure 14. 

These hierarchical droplets sequences strung on a polymer string have been identified in PEO, PAN, and PEM solutions. This type of instability differs from classical Rayleigh–Plateau pinching [67,71]. The formation of satellite droplets upon thinning of the thread formed by Oldroyd B liquid was studied in ref. [86,89]. Nevertheless, the proposed recursive relationship between filament diameters for successive generations in [86] does not fit correctly with the experimental data [112,113]. Numerical simulation of Bhat et al. [95] revealed the decisive role of inertia in the formation of satellite droplets. In the above theoretical works, the liquid was considered as a homogeneous medium, which does not allow a sufficient description of blister instabilities in polymer filaments. It is important that polymer solutions are characterized by concentration inhomogeneities, which under certain conditions, can grow and lead to separation into a solvent and a polymer-rich phase. Several mechanisms have been proposed to account for this effect. 

One of the mechanisms is based on the flow-induced phase separation of the polymer solution into a polymer-rich phase and a solvent-rich phase [121,122,123]. This is due to the dependence of the interaction energy of macromolecules on their conformation, in particular, on their orientation parameters. The interaction energy of semiflexible chains in solution at the third virial approximation is given by $f_{\mathrm{int}}=\frac{1}{2}B_{2}c^{2}+\frac{1}{3}B_{3}c^{3}$, where c is the concentration of the polymer segments and $B_{2}$ and $B_{3}$ are the second and the third virial coefficients, respectively. Within standard approximation, the second virial coefficient includes the contribution from the steric repulsion and van der Waals attraction (i.e., $B_{2}≃\frac{\pi }{2}l_{1}^{2}dk$, where $k=I\left(s\right)-\frac{\Theta }{T}$ and Θ is Θ-temperature). The function I(s) takes the steric repulsion between the segments into account and strongly depends on their orientation parameters. The third virial coefficient is $B_{3}≃\frac{3\pi^{2}}{32}l^{3}d^{3}I(s)$ [121,122,123]. The steric repulsion between the extended chains in the EC regime decreases with an increase in their orientation, therefore the balance between repulsive and attractive interactions is shifted toward attractions as the Weissenberg number $\mathrm{Wi}=\dot{\varepsilon }\tau$ increases. The polymer/solvent phase separation occurs when the second virial coefficient $B_{2}<0$ or $k=I\left(s\right)-\frac{\Theta }{T}<0$. The volume fraction of the polymer in the polymer-rich phase $\phi_{c}$ is obtained from the equality of osmotic pressure to zero: $\phi_{c}≃\left|k\right|/I(s)≃T\left|k\right|/\Theta$, $\phi_{c}\gg \phi$ when |k|≪1. The kinetics of phase separation was analyzed in ref. [115,116]. On the first stage of spinodal decomposition, the oriented domains with characteristic size $\xi \;\sim {\left(\frac{ld}{\phi \left|k\right|}\right)}^{1/2}$ in the cross-section of the thread are formed which then collapse laterally with the formation of a network of highly-oriented and stiff fibrils having diameter $d_{f}∼{\left(ld\right)}^{1/2}/\left|k\right|$, $d_{f}<<\xi$, and the longitudinal size $\xi_{z}\;\sim \frac{d}{\phi \left|k\right|}$. On the final stage, the network of fibrils then tends to compress by squeezing out the solvent to the surface. The characteristic time of the first two stages $t_{c}\sim \tau_{R}{\left(\frac{d}{\phi L\left|k\right|}\right)}^{2}$ is much shorter than $\tau_{R}$, therefore the phase separation could fully develop during the stretching regime. The formed annular solvent layer is unstable with respect to undulations [124], which should lead to the appearance of droplets. Methods of molecular dynamics modeling also confirm the formation of fibrillar structures by elongated PEO oligomers in an aqueous solution due to a decrease in the number of hydrogen bonds between PEO and water [125,126].

As mentioned above, the pearling structures are often observed after the elasto-capillary regime for PEO solutions [78,112,113,114,115,116]. Deblais, et al. [116] showed that temperature significantly affects the dynamics of thread thinning and the onset of pearling instability, which confirms the idea of phase separation. The period of the pearling structure is close to the period of the droplet structure that occurs when a filament of an inviscid liquid breaks: $\lambda ≃2\pi \sqrt{2}a_{0}$ [115]. A close value was also obtained in the analysis of the instability of a thin annular solvent layer on a wire [124]. 

Another mechanism leading to the instability of polymer solution thread is related to chain migration into thinner regions with a higher concentration due to the (SCC) stress–concentration coupling effect [127]. However, the SCC theory [128,129,130] does not predict flow-induced phase separations in unentangled polymer solutions. In the case of an extensional flow, the SCC effect is always much weaker than the flow-induced thermodynamic interaction effect [131].

Recently, a capillary mechanism for the formation of annular droplets in the TVC regime was proposed [107,131,132,133]. It occurs when the radius of the thread is smaller than the macromolecular contour length *L*. Such a mechanism was considered both in threads of solutions of rodlike macromolecules [131,132], where the droplet formation was found to be an activated process, and in threads of dilute solutions of semi-flexible polymers, where the droplet formation proceeds without any energy barrier [107,133]. In the last system, the solvent droplets are formed spontaneously as a hierarchical process when new solvent beads are constantly emerging on the polymer strings connecting the existing droplets during capillary-induced thinning of the polymer core and the string radius decreases linearly with time. The resulting highly polydisperse system of droplets is characterized by a self-similar (fractal) size distribution. This picture agrees with experimental observations concerning pearling instabilities and blistering patterns. The capillary mechanism of the pearling instability may be important for PAM solutions whose thinning does not depend on temperature, in contrast to PEO solutions [116]. The contour length of the PAM chains used in the experiment is *L* ~ 80 μm (the monomer length is $l_{1}\approx 0.4\;\mathrm{nm}$ and M_w_ ~ 15 × 10^6^ g/mol), so the critical radius should be on the order of or less than 8 μm, which is consistent with experimental data [116].

### 3.4. Stretching a Polymer Solution Jet by an External Load 

Fiber formation usually occurs by pulling a stream of polymer solution flowing out of the nozzle, Figure 13. Let us assume that the force $F_{ext}$ stretching the jet is localized at the take-up device. If the polymer solution flowing out of the nozzle of radius $a_{0}$ with the flow rate Q, then Equation (6) in the stationary regime of flow can be written as

(19) $$\frac{d}{dz}\left[-\gamma a+a^{2}\left(\rho v_{z}^{2}-\Sigma_{zz}+\Sigma_{rr}\right)\right]=0,\;\Sigma_{zz}-\Sigma_{rr}=3\eta_{s}\frac{dv_{z}}{dz}+\Sigma_{p}$$

where the flow velocity inside the jet is $v_{z}=\frac{Q}{\pi a^{2}}$. The boundary condition at the end of the jet (z=H, $a(H)=a_{H}$) implies the balance between the applied force and the jet tensile force:

(20) $$\pi a_{H}^{2}\left(\gamma /a_{H}+\Sigma_{zz}-\Sigma_{rr}\right)=F_{ext}$$

Integration of Equation (19) using (20) yields

(21) $$\frac{6Q\eta_{s}}{\pi a}\frac{da}{dz}-a^{2}\Sigma_{p}=\gamma a+\frac{\rho Q^{2}}{\pi^{2}}\left(\frac{1}{a_{H}^{2}}-\frac{1}{a^{2}}\right)-\frac{F_{ext}}{\pi }$$

Omitting the inertia and assuming $F_{ext}\gg 2\pi a_{0}\gamma$ in the case of Newtonian liquid ($\Sigma_{p}=3\eta_{p}\frac{dv_{z}}{dz}$) we obtain an exponentially decaying jet profile: $a\left(z\right)=a_{0}exp\left(-\frac{zF_{ext}}{6\pi Q\left(\eta_{s}+\eta_{p}\right)}\right)$. If the stretching of the jet occurs in the elastic regime when the polymer axial stress is dominant, $\Sigma_{p}>G$, then Equation (21) is reduced to $\Sigma_{p}=F_{ext}/(\pi a^{2})$. Since the axial stress is $\Sigma_{zz}^{p}≃\Sigma_{p}$, the jet profile is found from Equations (16) where $\frac{d\Sigma_{zz}^{p}}{dt}{=v}_{z}\frac{d\Sigma_{zz}^{p}}{dz}$ and $\dot{\varepsilon }=\frac{dv_{z}}{dz}=-\frac{2Q}{\pi a^{3}}\frac{da}{dz}$: $a(z)=a_{0}{\left(1+\frac{\pi a_{0}^{2}z}{Q\tau }\right)}^{-1/2}$ [120]. Thus, the thinning of the jet occurs according to a power law. In this case, the Weissenberg number is constant along the jet, Wi=1 $\left(\dot{\varepsilon }=\tau^{-1}\right)$, and the orientational parameter of the chains increases as $s=R_{z}/L∼s_{0}{\left(\frac{F_{ext}z}{Q\eta_{p}}\right)}^{1/2}$(*s* ≲ 0.5). This means that the steric repulsion between the chains decreases and spinodal decomposition of the solution with the release of the solvent is possible. Such an effect was observed with PAN solutions [120]. 

### 3.5. Effect of Gravity 

The shape of a falling jet and the critical length before its disintegration into drops were the subjects of long-term interest. The length of a falling get can be very long [134] and greatly exceed the limit predicted by the Rayleigh–Plateau values [135]. The general explanation connects this effect to the transition from the capillary dominating regime of flow to the viscous regime. Clarke presented the complete formulation of the dynamic equation for the shape of a falling jet formed by a Newtonian liquid [136,137]. The validity of the general solution obtained by Clarke was examined rather carefully in [138] for the micro-flow device where a jet is formed between a feeding capillary and a suction cell. The results of studying the gravitational flow in a wide Reynolds number range confirmed the validity of the universal solution to the dynamic problem. The formation of a stable jet happens after the transition from periodic dripping to jetting along with increasing the velocity of a fluid [139]. Theoretical analysis of the behavior of the free-falling viscoelastic liquid jet allowed for establishing the instability boundary connected with the influence of surface effects [140,141]. The shape of free-falling stable jets created by viscoelastic concentrated polyacrylonitrile solutions were studied in [142], where the superposition of viscoelastic, capillary, and inertial forces for fluids with different rheological properties were analyzed. At a low polymer concentration, the jet profile is determined by the balance of capillary, inertial, and gravitational forces, while at higher velocities and highly viscous solutions, the balance of viscous, inertial, and gravitational forces becomes dominant. At very high concentrations, the role of elasticity increases, but the Weissenberg number remains below the critical value corresponding to the unfolding of polymer chains [142].

### 3.6. Electrospinning

**Figure 15:**
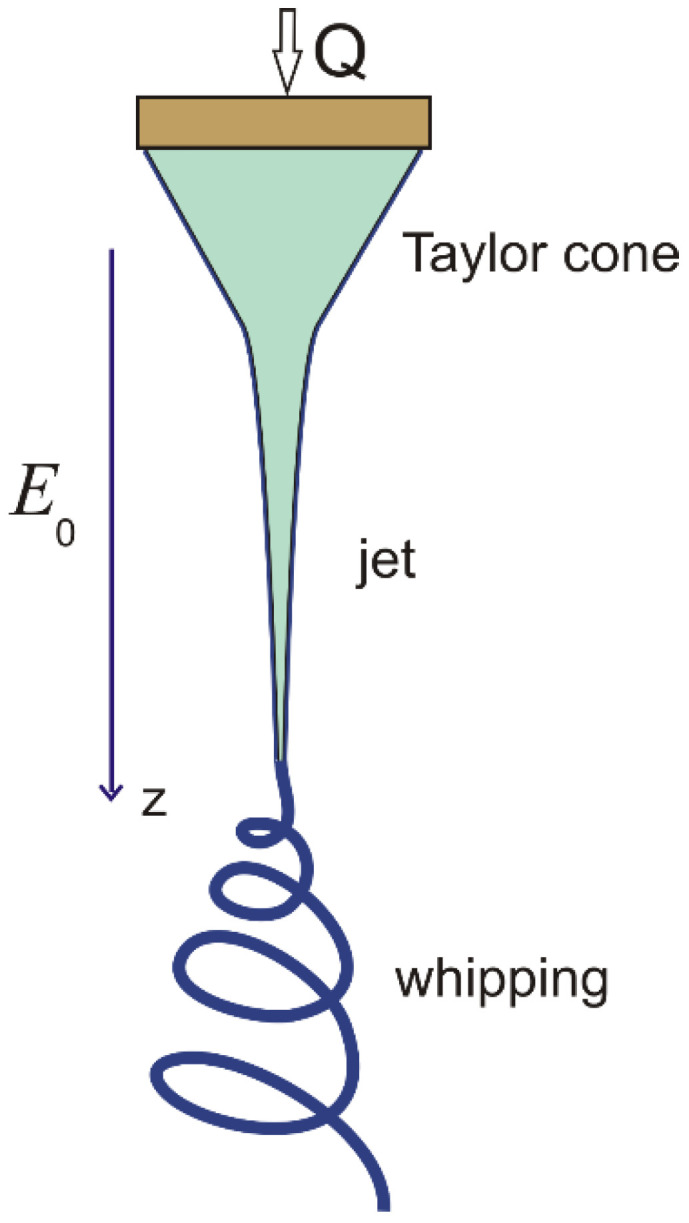
A typical electrospinning jet pattern includes a Taylor cone, a straight jet, and a whipping jet. Authors’ drawing.

The driving force of the jet flow in this case is of an electrostatic nature. Experiments show that if a voltage exceeding a critical value is applied to the meniscus of a liquid, it assumes a conical shape, also known as a Taylor cone, the top of which emits a thin jet [143,144,145], Figure 15. 

Taylor was the first to show that perfectly conducting liquid forms a conical shape with the apex semi-angle $\theta_{T}=49.3^{\circ }$ due to a balance between the electrostatic and capillary forces [143]. The conical surfaces were also predicted for the ideal dielectric liquids whose dielectric constant ε exceeds some critical value $\varepsilon >\varepsilon_{c}\approx 17.6$ [146]. The cone half-angle θ in this case depends on ε and varies in the range $0<\theta <49.3^{\circ }$. The surface of the dielectric cone carries only the polarization charge and the emanation of the jet from the cone apex is impossible similary to the case of the perfectly conducting liquid forming the Taylor cone. Recently, self-similar conical structures different from the hydrostatic Taylor cone and capable of emitting charges were described [62,147,148,149,150,151]. There are two families of micro-cones carrying surface charges [62]. The first family constitutes needle-like micro-cones having a small apex angle, $0<\theta_{c}^{}\left(\varepsilon \right)<27^{\circ }$, ε>1, and $\theta_{c}^{}\left(\varepsilon \right)\to 0$ at ε→∞. The micro-cones from the second family appear at ε>12.6 and have the apex angles $36^{\circ }<\theta_{c}^{}\left(\varepsilon \right)<49.3^{\circ }$ where the upper boundary corresponds to the Taylor value $2\theta_{c}^{}=98.6^{\circ }$ (at ε→∞). Based on the Onsager principle, it was shown that needle-like micro-cones are more stable [62,148]. 

The behavior and the shape of the electrospinning jets were widely studied experimentally [152,153,154,155,156]. For the theoretical analysis of the shape of rectilinear jets, the slender body approximation is often used. The force balance Equation (7) for a stationary jet carrying only surface charge is written as

(22) $$\frac{d}{dz}\left[a^{2}\left(\rho v_{z}^{2}+\Sigma_{rr}-\Sigma_{zz}\right)-\gamma a\right]≃2aF_{\tau }+a^{2}\frac{dF_{n}}{dz}$$

The electrostatic field inside the jet is determined from equation [146]

(23) $$E_{z}≃E_{0}-ln\frac{1}{\left|a_{z}^{’}\right|}\left(\frac{1}{\varepsilon_{0}}\frac{d\left(a\sigma_{f}\right)}{dz}-\frac{\left(\varepsilon -1\right)}{2}\frac{d^{2}\left(E_{z}a^{2}\right)}{dz^{2}}\right)$$

Here, $E_{0}$ is the external field generated by the electrode, $\varepsilon_{0}$ is the dielectric permittivity of the vacuum, and $\sigma_{f}$ is the density of the free charges on the jet surface. The polarization charge is $\sigma_{p}≃-\frac{\left(\varepsilon -1\right)\varepsilon_{0}}{2a}\frac{d\left(E_{z}a^{2}\right)}{dz}$, so the normal component of the electric field is $E_{n}=\frac{\sigma_{p}}{\left(\varepsilon -1\right)\varepsilon_{0}}$. The flow inside the jet is characterized by the average velocity $Q/(\pi a^{2})$, where Q is the volume of liquid which is issued from the nozzle per unit of time. The electric current inside the jet is a sum of the bulk current $I_{b}$, convective current $I_{Q}$, and surface current $I_{s}$: $I=I_{b}+I_{Q}+I_{s}$ where $I_{b}≃\pi a^{2}KE_{z}$ (K is the bulk conductivity of the liquid), $I_{Q}≃\frac{2Q\sigma_{f}}{a^{2}}$, and $I_{s}≃2\pi a\sigma_{f}\mu E_{z}$ (μ is the mobility of the surface ions) [62]. At high flow rates, the surface current $I_{s}$ can be neglected. Different electrospinning regimes of the Newtonian liquid were studied numerically [157,158,159,160]. The asymptotic shape of the jet is determined by the balance of inertial and electrical forces: $a\left(z\right)={\left(\frac{\rho Q^{3}}{2\pi^{2}IE_{0}z}\right)}^{1/4}$ [161]. The effect of polymer elasticity on the jet profile with using Oldroyd-B and FINE-P models was considered by Carroll and Joo [162]. However, only low Weissenberg numbers were considered. Experiments show that the extension rate in the cone/jet transition region exceeds the inverse chain relaxation time (Wi>1) which results in unfolding of the polymer chains, such that they are stretched inside the jet [155,163,164].

At relatively low flow rates, $Q\tau_{E}\ll D^{3}$, where D is the characteristic size of the meniscus and $\tau_{E}=\frac{\varepsilon \varepsilon_{0}}{K}$ is the charge relaxation time, the meniscus takes a conical shape and its stability is mainly determined by the balance of electrostatic and capillary forces, $F_{n}=\frac{\varepsilon \varepsilon_{0}E_{\tau }^{2}}{2}+\frac{{\left(\sigma_{f}+\sigma_{p}\right)}^{2}}{2\varepsilon_{0}}\sim \frac{\gamma }{a}$, and the surface charge density is estimated as $\sigma_{f}∼{\left(\gamma \varepsilon_{0}/a\right)}^{1/2}$ [165]. The electric field inside the cone/jet transition zone of radius *a*_0_ is mainly generated by the surface charges of the cone, $E_{z}\sim {\left(\frac{\gamma }{\varepsilon_{0}a_{0}}\right)}^{1/2}$, whereas on a distance z≫D, $E_{z}\;⋍\;E_{0}$. If $E_{0}\ll {\left(\frac{\gamma }{\varepsilon_{0}a_{0}}\right)}^{1/2}$, the electric field in the entire region z>0 can be approximately represented as the sum $E_{z}⋍E_{0}+\kappa {\left(\frac{\gamma }{\varepsilon_{0}\left(a_{0}+z\right)}\right)}^{1/2}$, where κ is a numerical factor which depends on the geometry of the cone. The bulk current dominates inside the meniscus, whereas in the jet it is determined by the convective current $I_{Q}$. In the transition zone $I_{b}⋍I_{Q}⋍I/2$. From here we find the radius of the transition zone $a_{0}∼{\left(Q\varepsilon_{0}/K\right)}^{1/3}$ and the electric current $I∼{\left(\gamma KQ\right)}^{1/2}$. 

Next, let us focus on the regime when the Weissenberg number Wi≳1 in the cone/jet transition zone. In this case, the polymer chains are stretched inside the jet and the stress difference can be written as $\sum_{zz}-\sum_{rr}⋍\sum_{0}\frac{a_{0}^{4}}{a^{4}}$ [166] where $\sum_{0}≳$*G* is the stress in the cone/jet transition zone. Substitution of this formula in Equation (22) and integration yields ($z\gg a_{0}$)

(24) $$\left(\frac{\rho Q^{2}}{2\pi^{2}}-\frac{\sum_{0}a_{0}^{4}}{2}\right)\left(\frac{1}{a^{4}}-\frac{1}{a_{0}^{4}}\right)+\gamma \left(\frac{1}{a}-\frac{1}{a_{0}}\right)⋍\frac{I}{Q}\left(E_{0}z+2\kappa \sqrt{\frac{\gamma z}{\varepsilon_{0}}}\right)$$

This equation facilitates recovery of the main asymptotes of the jet profile which were found experimentally [154,156]: $a\left(z\right)\propto z^{-1/2}$ when $a_{0}\ll \;z\ll D$ and $a\left(z\right)\propto {\left(z+z_{0}\right)}^{-1}$ where $z_{0}\sim D\sqrt{\frac{\gamma }{\varepsilon_{0}E_{0}^{2}D}}$ when z≫D; $a\left(z\right)\propto {\left(z\right)}^{-1/4}$ at z→∞ and $\frac{\rho Q^{2}}{2\pi^{2}}>\frac{\sum_{0}a_{0}^{4}}{2}$. It is interesting to note that for low flow rates when $\frac{\rho Q^{2}}{2\pi^{2}}<\frac{\sum_{0}a_{0}^{4}}{2}$, the straight jet has a finite length $\sim \frac{Q\gamma }{IE_{0}a^{*}}\;$, where $a^{*}\sim {\left(\pi^{2}\sum_{0}a_{0}^{4}-\rho Q^{2}\right)}^{1/3}\gamma^{-1/3}$. A similar result was obtained in numerical calculations [166,167]. In this case, the orientation parameter increases along the jet axis as $s⋍s_{0}a_{0}^{2}/a^{2}$, and reaches its maximum value at the end of the straight section of the jet. For z>H, the behavior of the chain should be unstable. The length of the rectilinear section of the jet increases with an increase in the flow rate and decreases with an increase in the field strength, which agrees with the experiment [168,169]. For large flow rates, $\frac{\rho Q^{2}}{2\pi^{2}}>\frac{\sum_{0}a_{0}^{4}}{2}$, the jet is rectilinear. In this mode, the order parameter changes non-monotonically along the jet: first, it increases up to a certain maximum value (at which the Weissenberg number reduces to Wi∼1) and then decreases [166,167]. The decrease is associated with the relaxation of the polymer chains. 

The formation of fibers from the jet occurs as a result of the chain orientation, aggregation, and evaporation of the solvent. Numerical calculations have shown that during electrospinning, polymer chains can be strongly elongated along the flow in the rectilinear section of the jet, so that their orientational order parameter reaches the value s≳0.5 [166,167]. The high orientation of polymer chains can lead to a phase separation of the polymer solution [166] with the emergence of string-like structures. These structures were identified experimentally [170,171].

With a further decreasing flow rate, the convective current decreases and therefore, another regime with $Q<\mu_{+}Eb^{2}$ is realized. The cone/jet transition is determined by the equality of the bulk and surface currents, $I_{b}≃I_{s}$. The radius of the transition zone here is $b=b_{3}∼{\left(\mu_{+}/K\right)}^{2/3}{\left(\gamma \varepsilon_{0}\right)}^{1/3}$. The surface current is dominated at $b\ll b_{3}$ as well as the meniscus issued needle-like micro-cones in this case [148]. These micro-cones were identified in near-field electrospinning and can be used to create a nanoscale fiber [172].

## 4. Conclusions

Elasticity is the immanent property of polymers due to the flexibility and anisotropy of macromolecular chains. We discussed the role of elasticity in different flow modes of polymeric liquids (solutions and melts) considering both sides of the story: macroscopic effects and the input of elasticity into the formulation and solution of basic dynamic equations of a continuum medium. In all cases, the Weissenberg number (Wi) is a crucial factor in determining the liquid-to-solid-like type of polymer behavior (at Wi ~ 1). 

We have considered the general picture of the flow of elastic liquids associated with their elasticity through short capillaries. The effects of the inlet vortex and the die swelling are well known. The relaxation phenomenon determines apparent high values of end correction in the capillary flow. Then, the role of the length of a capillary is related to the duration of relaxation. The hydrodynamic resistance of short capillaries is the universal function of the Deborah number. The die swelling also depends on the capillary length due to the partial relaxation and swelling decrease for long capillaries in comparison with short ones. In addition to the initiation of secondary flows, the Weissenberg number is also responsible for the transition to the elastic instability of the stream inside the capillary and the periodic oscillation of the jet at the capillary exit. Indeed, both effects are observed at Wi ≳ 1.

Elasticity also affects the shape of the jet leaving the capillary. However, examination of the behavior of jets should be based on the analysis of fundamental dynamic equations which requires taking into account the other factors such as the gravity force, surface tension, and finally the electric forces in the case of electrospinning. The theoretical analysis correlated with the experimental fact showed that the polymer elasticity becomes dominating at high extension rates (Wi ≳ 1) when the polymer coils unfold. In this case, the nature of the extension force plays an important role. The Weissenberg number correlates with the transition from the viscous flow of the jet to its solid-like behavior.

## Data Availability

Not applicable.
